# Two-stepped majority voting for efficient EEG-based emotion classification

**DOI:** 10.1186/s40708-020-00111-3

**Published:** 2020-09-17

**Authors:** Aras M. Ismael, Ömer F. Alçin, Karmand Hussein Abdalla, Abdulkadir Şengür

**Affiliations:** 1 Sulaimani Polytechnic University, Sulaymaniyah, Iraq; 2 Electrical Engineering Department, Engineering and Natural Sciences Faculty, Malatya Turgut Ozal University, 44210 Malatya, Turkey; 3 Psychology Department, Raparin University, Ranya, Iraq; 4 Electrical-Electronics Engineering Department, Technology Faculty, Firat University, Elazig, Turkey

**Keywords:** EEG-based emotion recognition, EEG rhythms, Wavelet packet entropies, Fractal dimensions, Majority voting

## Abstract

In this paper, a novel approach that is based on two-stepped majority voting is proposed for efficient EEG-based emotion classification. Emotion recognition is important for human–machine interactions. Facial features- and body gestures-based approaches have been generally proposed for emotion recognition. Recently, EEG-based approaches become more popular in emotion recognition. In the proposed approach, the raw EEG signals are initially low-pass filtered for noise removal and band-pass filters are used for rhythms extraction. For each rhythm, the best performed EEG channels are determined based on wavelet-based entropy features and fractal dimension-based features. The k-nearest neighbor (KNN) classifier is used in classification. The best five EEG channels are used in majority voting for getting the final predictions for each EEG rhythm. In the second majority voting step, the predictions from all rhythms are used to get a final prediction. The DEAP dataset is used in experiments and classification accuracy, sensitivity and specificity are used for performance evaluation metrics. The experiments are carried out to classify the emotions into two binary classes such as high valence (HV) vs low valence (LV) and high arousal (HA) vs low arousal (LA). The experiments show that 86.3% HV vs LV discrimination accuracy and 85.0% HA vs LA discrimination accuracy is obtained. The obtained results are also compared with some of the existing methods. The comparisons show that the proposed method has potential in the use of EEG-based emotion classification.

## Introduction

Emotions are defined as biological situations associated with the nervous system [1]. Neuro-physiological variations associated with thoughts, feelings, behavioral responses and a degree of pleasure or displeasure are the causes of emotions. Emotions have an important role in communication and facial expressions are the main indicators of emotions. Besides facial expressions, human sound, and body gestures are known to be the other important emotion indicators [1]. Recently, the use of electroencephalogram (EEG) in emotion detection has been increasing. The EEG signals reveal the electrical status of the brain while having different emotions. Besides image processing-based emotion analysis, the recent trend goes thought the EEG-based emotion analysis.

Chao et al. [1] proposed a framework, which was based on deep learning, for EEG-based emotion recognition. The proposed framework was based on multiband feature matrix and capsule networks. Frequency domain- and frequency band-based characteristics were used to construct the multiband feature matrix. The capsule network was used in the classification stage of the proposed work. The authors used the DEAP dataset in their experiments and reported that the proposed method outperformed most of the existing methods. Koelstra et al. used frequency-domain features, which were extracted from the power spectrum density, for EEG-based emotion recognition [2]. The Gaussian Naïve Bayes classifier was used to classify the EEG-based emotions into binary classes such as valence and arousal. The DEAP dataset was used in experiments and promising results were obtained. Alazrai et al. proposed a methodology for EEG-based emotion recognition [3]. The authors used a new time–frequency (TF)-based features for efficient classification. The quadratic TF transformation was used and 13 TF-based features were extracted. The Support Vector Machines (SVM) classifier was used in classification. The experiments that were carried out on the DEAP dataset produced accuracy scores in the range of 73.8% and 86.2%. Huang et al. developed a novel approach for EEG-based emotion recognition [4]. The developed approach was based on asymmetric spatial patterns that were extracted from some pairs of spatial filters. Experiments were carried out for discrimination of the arousal and valence status and satisfactory results were reported by the authors. Candra et al. used the wavelet transform for EEG-based emotion classification. The authors used the entropy of the wavelet coefficients as features and the SVM classifier was used to classify them into valence and arousal classes [5]. The authors also mentioned that the efficiency of the EEG-based emotion classification could be improved using shorter time segments of the EEG signals. The experiments handled by the authors released that using the shorter time segments of the EEG signals increased the classification accuracy. Rozgiç et al. developed a three-stepped approach for EEG-based emotion classification [6]. The authors initially used an overlapping window for segment-level feature extraction. The obtained segment-level features were transformed into the response-level features using a novel non-parametric nearest-neighbor model. Finally, the transformed features were classified in the third step of the proposed method. The authors used the DEAP dataset and reported satisfactory results. Al-Nafjan et al. used power spectral density and frontal asymmetry features for human emotion classification [7]. The authors opted to use deep neural networks (DNN) in the classification of the extracted features. The EEG signals from the DEAP dataset were used in experiments and the benefit of the DNN for the large dataset was reported. Chen et al. proposed deep convolutional neural networks (DCNN) for EEG-based emotion recognition [8]. The authors developed a CNN model and trained it in the end-to-end fashion. Authors also opted to extract the time and frequency domain features and used machine learning techniques to compare their results with the traditional approaches. The DEAP dataset was used in experiments and the ROC curve was used in performance evaluation. Zhang et al. proposed the ontology-based approach for EEG-based emotion recognition [9]. The developed ontology-based system depended on two bases. The first basis covered a model that considers the users’ contexts, EEG data and the environmental situations, and the second one was related to modeling of the reasons on users’ emotions. The DEAP dataset was used in experiments and accuracy scores 75.19% and 81.74% were reported for valence and arousal classes, respectively. Atkinson et al. proposed a novel feature-based approach for EEG-based emotion recognition [10]. In the proposed approach, the authors used statistical feature selection methods in an ensemble way to increase the efficiency of the extracted features. The SVM classifier was used at the classification stage of the work and the authors mentioned that the proposed method outperformed other machine learning methods. Tripathi et al. used deep learning for EEG-based emotion recognition [11]. More specifically, the authors explored two different deep neural networks models, one was a simple DNN and the other was a CNN model, for emotion classification. The carried-out experiments showed that the deep models outperformed other state-of-the-art methods. Zhong et al. proposed a methodology for EEG-based emotion recognition [12]. The proposed method employed a multiple-fusion-layer-based ensemble classifier of stacked auto-encoder. Each of the stacked auto-encoder consisted of three hidden layers. An additional deep model was considered to achieve the ensemble structure. For feature fusion, an adjacent graph-based network model was used. The fused features were fed into the classification of the emotion into arousal or valence states. Zhuang et al. used empirical mode decomposition (EMD) for EEG-based emotion detection [13]. After obtaining the intrinsic mode functions, energy and phase information were extracted to use as features for the characterization of the emotions. The SVM classifier was used on the DEAP dataset and satisfactory results were reported. Li et al. achieved EEG-based emotions recognition that was based on an ensemble of SVM classifiers [14]. A weighted fusion scheme was considered to construct the ensemble structure. Moreover, a channel division approach was employed that was based on neuropsychological theory, to acquire the information from different areas of the brain. The DEAP dataset was used in the experiments and high performance was reported by the authors. Zhang et al. used EMD and sample entropy for classifications of emotion EEG signals into valence and arousal classes [15]. The two channels of the EEG signals were used to convert them into intrinsic mode functions. The first four intrinsic mode functions were considered to extract the sample entropy features. The SVM classifier was used in the recognition step of the proposed study. The proposed method achieved a 94.98% accuracy score for binary-class tasks on the DEAP dataset.

**Fig. 1:**
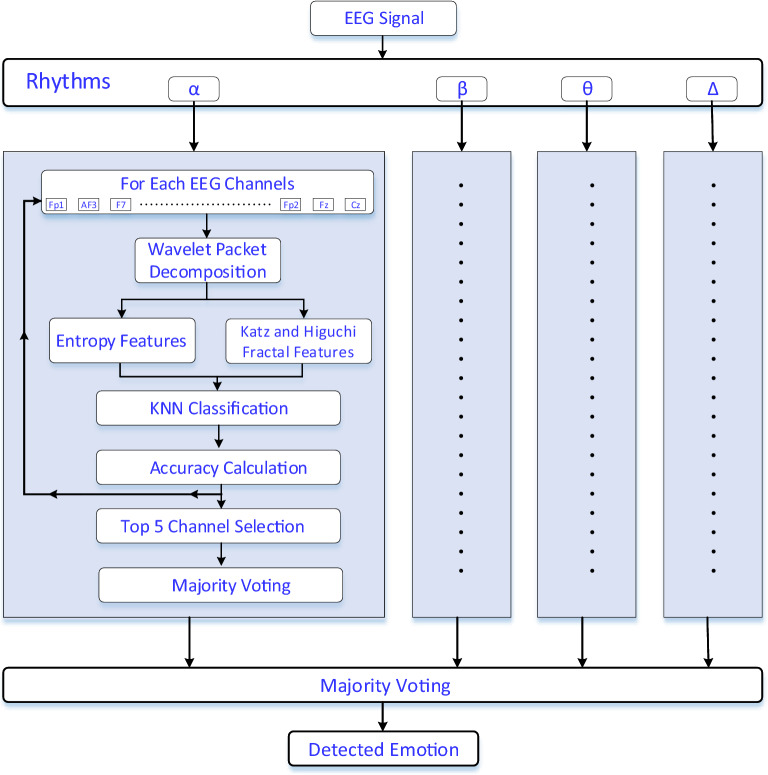
The graphical illustration of the proposed work

In this paper, an efficient two-stepped majority voting approach is proposed for EEG-based emotion recognition. The proposed approach is quite simple and easy to implement. The input EEG signals are initially low-pass filtered for noise removal. And, the EEG rhythms are extracted using the band-pass filters. For each rhythm, the effective channels of the EEG signal are investigated. The top 5 EEG channels are considered and the rest are discarded. The channel’s achievements are detected using a procedure that is shown in Fig. 1. For each rhythm, from all EEG channels, wavelet-based entropy features such as “Shannon”, “Logarithmic energy”, “Threshold”, “Sure” and “Norm” are calculated. For wavelet decomposition, wavelet packet decomposition is considered. Wavelet packet decomposition employs decomposition procedure not only approximation coefficients but also the detail coefficients, respectively. Thus, the interesting knowledge in the details is also revealed. Two fractal dimension features, namely Katz and Higuchi are also considered in feature extraction. Thus, the number of features for each sample is based on the level of the wavelet packet decomposition. The KNN classifier is used in the classification of the obtained features into emotion labels. The KNN is selected due to its simplicity. In KNN, the number of the nearest neighbor was set to three and Minkowski distance was used for distance metric. These values were obtained heuristically during the experimental processes. The first majority voting procedure is applied on each rhythm on the best channels predictions and the second majority voting procedure is applied to all rhythms predictions. Thus, an aggregated prediction is obtained. The DEAP dataset is used in experiments and classification accuracy, sensitivity and specificity scores are calculated for performance measurements. The experiments are carried out to classify the emotions into two binary classes such as high valence (HV) vs low valence (LV) and high arousal (HA) vs low arousal (LA). The experiments show that 86.3% HV vs LV discrimination accuracy and 85.0% HA vs LA discrimination accuracy is obtained. The contributions of this paper are;

1. Two-stepped majority voting approach is proposed for efficient EEG-based emotion recognition.
2. Comprehensive experimental works are conducted on EEG rhythms vs EEG channels to detect the efficient channels and rhythms of the EEG signals and with majority voting effective results are obtained.

The remainder of this paper is as follows. In the next section, the proposed method and the background theories are described. In Sect. 3, experimental works and obtained results are examined. In the last section, conclusions, discussions and future works are described.

## Proposed method

Figure 1 shows the graphical illustration of the proposed work. The input EEG signals are initially low-pass filtered for the elimination of the noises. The pass band and stop band frequency values were set to 0.15 and 0.2 Hz, respectively. In addition, pass band ripple and stop band attenuation values were set to 1 and 60 dB, respectively. After noise elimination, the rhythm of the EEG signals is extracted and all procedures are repeated for each rhythm. The band-pass filters are used to acquire the alpha, beta, theta and delta rhythms. Alpha rhythm covers the 8–12-Hz frequency range. Beta rhythm is in the range of 12–30 Hz. Theta and delta rhythms are in the range of 4–8 Hz and 0–4 Hz, respectively. As the best five channels are seeking, for all channels, the wavelet packet decomposition is applied and the entropy and fractal dimension features are extracted from the obtained wavelet coefficients. These features are the entropies namely “Shannon”, “Logarithmic energy”, “Threshold”, “Sure” and “Norm” and Katz and Higuchi fractal dimensions. k-NN classification is applied to get the channels predictions. And, the accuracy score is calculated to get the channels’ achievements. The best 5 channels’ predictions are used in majority voting to get the rhythm’s prediction. After obtaining the majority voting predictions of all rhythms, the second majority voting procedure is applied to the rhythms’ predictions. The obtained final prediction is used as the output of the proposed method.

### Wavelet packet transform and related entropies

Discrete wavelet transform (DWT) presents time–frequency information about the signal. Wavelet Packet Transform (WPT) is an improved version of the DWT. Because more filters are applied to the signal *x*(*t*) compared to DWT. The DWT is passed the previous approximation coefficients through Quadrature Mirror Filters (QMF). In WPT, both approximation and detail coefficients are passed through QMF. This situation provides more information about the *x*(*t*) at different levels, consequently higher performance in classification problems. The WPT can be described as follows:

1 $$\left\{ {\begin{array}{*{20}l} {d_{0,0} \left( t \right) = x\left( t \right)} \\ {d_{i,2j - 1} \left( t \right) = \sqrt 2 \mathop \sum \limits_{k} h\left( k \right)d_{i - 1,j} \left( {2t - k} \right)} \\ {d_{i,2j} \left( t \right) = \sqrt 2 \mathop \sum \limits_{k} g\left( k \right)d_{i - 1,j} \left( {2t - k} \right),} \\ \end{array} } \right.$$

where *d*_*i,j*_ symbolize the coefficient of the WPT at the *i*th level for the *j*th node, *h*(*k*) is high-pass QMF and *g*(*k*) is low-pass QMF. As mentioned above, the features which are information about the raw signals are most important in classification problems [15–19]. Therefore, the WPT coefficients can be used for this purpose. Entropy measures uncertainty of signal, so it can be a useful tool in signal processing. Entropy can be calculated from the energy of the WPT coefficient [15, 18–20]. The Shannon Entropy (SE) can be calculated from Eq. (2) at the related node.

2 $${\text{SE}}_{i,j} = - \mathop \sum \limits_{k = 1}^{N} d_{i,j,k}^{2} { \log }\left( {d_{i,j,k}^{2} } \right).$$

The Logarithmic Energy Entropy (LEE) can be expressed at the related node in Eq. (3).

3 $${\text{LLE}}_{i,j} = - \mathop \sum \limits_{k = 1}^{N} log\left( {d_{i,j,k}^{2} } \right).$$

The Norm Entropy (NE) can be described at the related node as a following:

4 $${\text{NE}}_{i,j} = \mathop \sum \limits_{k = 1}^{N} \left| {d_{i,j,k} } \right|^{p} ,$$

where *p* is equal to or greater than one [20]. Threshold Entropy (TE) can be defined at the related node:

5 $${\text{TE}}_{i,j,k} = \left\{ {\begin{array}{*{20}c} {1,\quad \left| {d_{i,j,k} } \right| > T} \\ {0,\quad \left| {d_{i,j,k} } \right| \le T, } \\ \end{array} } \right.$$

6 $${\text{TE}}_{i,j} = \mathop \sum \limits_{k = 1}^{N} {\text{TE}}_{i,j,k} ,$$

where *T* is the threshold value. TE is equal to the sum of the instances in which the signal (| *d*_*i,j,k*_ |) is greater than the *T* [20]. The Sure Entropy (SUE) can be expressed at the related node as follows;

7 $${\text{SUE}}_{i,j} = \mathop \sum \limits_{k = 1}^{N} \hbox{min} \left( {d_{i,j,k}^{2} ,\varepsilon^{2} } \right)\;{\text{such}} \;{\text{that}}\; \left| {d_{i,j,k} } \right| \le \varepsilon ,$$

where *ε* symbolizes a positive threshold and must be equal or greater than two.

### Higuchi’s fractal dimension

The nonlinear quantifying of complexity dynamical time sequence can be calculated using Higuchi’s fractal dimension (HFD) [21, 22]). Suppose *x*(1), *x*(2),…, *x(N)* a discrete-time sequence with *N* length to be analyzed. The discrete signal can be constructed *k* new time sequence as: $X_{k}^{m} :x\left( m \right), x\left( {m + k} \right),x\left( {m + 2k} \right), \ldots ,x\left( {m + {\text{int}}\left[ {\frac{N - k}{k}} \right]k} \right)$ for *m *= 1,2,…,*k*. here, *m* symbolizes the initial time, *k* represents the time interval and int(.) is the integer part of a real number. For each of the *k* time sequence or $X_{k}^{m}$ curves; the curve length is described as follows:

8 $$L_{m} \left( k \right) = \frac{1}{k}\left[ { \left( {\mathop \sum \limits_{i = 1}^{a} \left| {x\left( {m + ik} \right) - x\left( {m + \left( {i - 1} \right)k} \right)} \right|} \right)\frac{N - 1}{ak}} \right],$$

where *a* is ${\text{int}}\left( {\frac{N - m}{k}} \right)$. In Eq. (8), the average length is calculated as the mean of the *k* lengths *L*_*m*_(*k*) for all *m*. This process is repeated to obtain *L*(*k*) the mean curve length for each *k* which ranged from 1 to free a parameter *k*_max_.

9 $$L\left( k \right) = \frac{1}{k}\left[ {\mathop \sum \limits_{m = 1}^{k} L_{m} \left( k \right)} \right].$$

HFD is expressed as a follows [22]:

10 $${\text{HFD}} = \frac{{\ln \left( {L\left( k \right)} \right)}}{{\ln \left( {1/k} \right)}};$$

the HFD may typically produce the value between 1 and 2 for EEG waveforms [21]

### Katz’s fractal dimension

Katz’s fractal dimension (KFD) is commonly used to measure the complexity of EEG signals. KFD is described as follows [23, 24]:

11 $${\text{KFD}} = \frac{{\log \left( {L/a} \right)}}{{\log \left( {d/a} \right)}},$$

where *L* is the total length of the time series, *a* symbolizes the average number of steps and *d* describes as the Euclidian distance between the first sample and the sample which provides farthest distance [23, 24].

### k-Nearest Neighbor classifier

In the among supervised machine learning methods, the k-Nearest Neighbor (k-NN) is widely used due to its simplicity and good performance [18]. k-NN classifier needs a training dataset which consists of positive and negative class. The class of a new sample is assigned according to the distance to the nearest training class. This process is dilated taking into consideration the nearest *k* points and by assigning the sign of majority. The number of neighbor *k* is commonly odd and small (e.g., 1, 3 or 5). However, larger *k* may provide to reduce noisy effects in the training set. The distance can be calculated with methods such as Euclidean, Minkowski, Chebyshev, Cityblock, etc. [18, 25, 26].

## Experimental works and results

A workstation configured with Intel(R) Xeon(R) CPU E5-1650 @3.60 GHz with 64 GB memory and the NVIDIA Quadro M4000 GPU was used in experiments. The codes were run on the MATLAB software. The DEAP database, which was used in experiments, contains 32-channel EEG and 8 peripheral physiological signals. 16 men and 16 women were used to collect the EEG signals. More specifically, 40 EEG signals for each subject were collected where 40 1-min length videos with different emotional tendencies were used. The subjects were asked to rate the watched videos on a scale of 1–9 in terms of valence, arousal, liking, and dominance. During the labeling of the emotions, binary labeling was considered. The valence ratings that were smaller than 5 were assumed to have negative emotions. And, valence ratings higher than 5 were considered to have positive emotions. Besides, the arousal rating scales show ranging from passive to active. The arousal ratings that were smaller than 5 were considered as passive and other rating scales that were higher than 5 were assumed as active. Thus, the obtained class labels were low valence (LV) vs high valence (HV) and low arousal (LA) vs high arousal (HA), respectively. The experiments were conducted in tenfold cross-validation fashion. The pseudo-code of the proposed study is given in Table 1. As seen in Table 1, the input of the code was raw EEG signals and the output was the detected emotions. For all subjects, the EEG signals were initially low-pass filtered and for each rhythm, the channel achievements were calculated and the top five successful channels were used in majority voting procedure to get the rhythm’s predictions. The number of five was selected heuristically during the experiments. These predictions were saved and in the end, the second majority voting was applied to the rhythms’ predictions to obtain the final prediction.

**Table 1:**
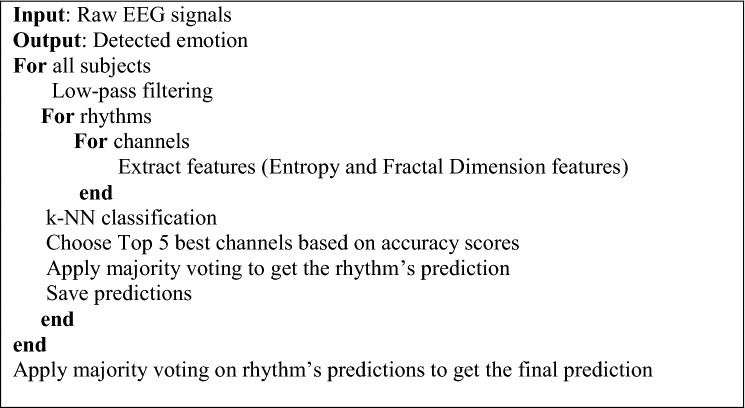
The pseudo-code of the proposed method

**Fig. 2:**
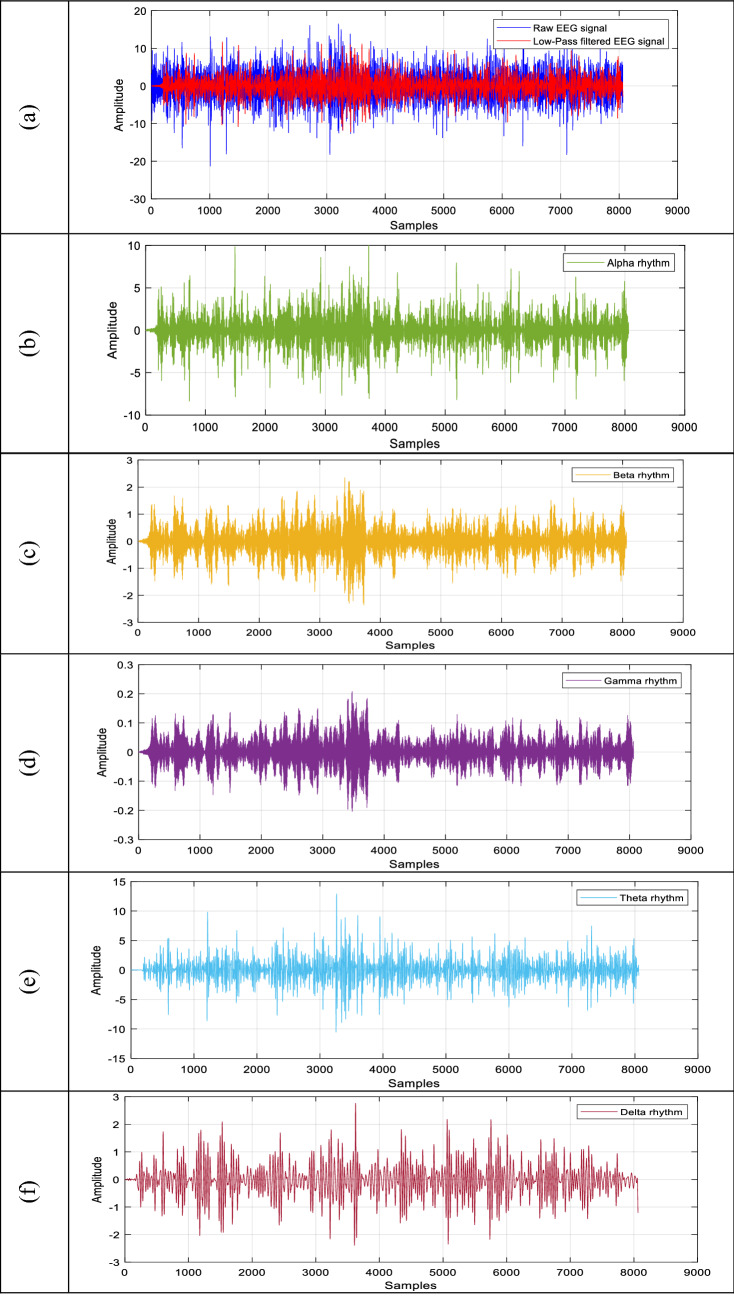
The rhythms of the EEG signal. **a** Low-pass filtered EEG signal, **b** Alpha rhythm, **c** Beta rhythm, **d** Gamma rhythm, **e** Theta rhythm, **f** Delta rhythm

As it was mentioned in the previous sections, the EEG signals were initially filtered with low-pass filtering to eliminate the noises. The alpha, beta, gamma, theta and delta rhythms of EEG signals were decomposed. Figure 2 shows the example of EEG signals and their corresponding rhythms. The sample EEG signal was acquired from the Fp1 electrode and its sampling frequency was 128 Hz. The EEG signal contains 8064 samples.

**Fig. 3:**
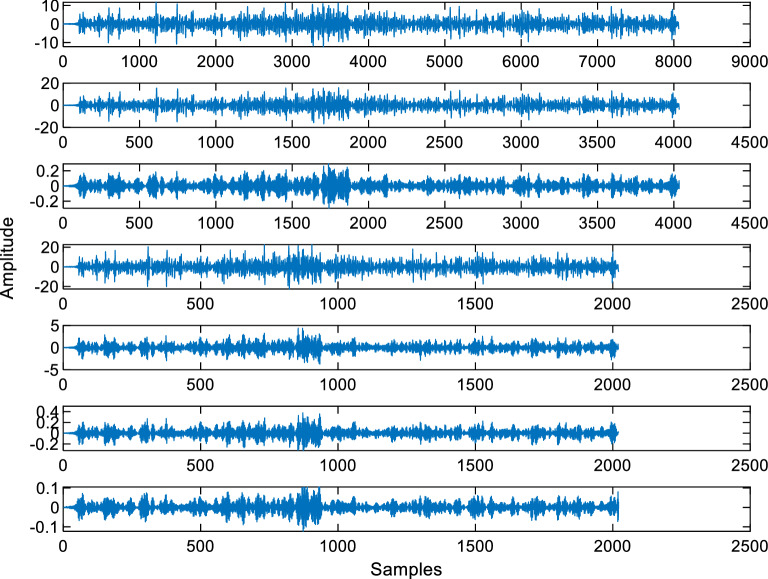
The wavelet packet decomposition of an EEG signal. 2 level decomposition is used using the Daubechies wavelet of order 4

While Fig. 2a shows the raw and low-pass-filtered sample EEG signal, Fig. 2b–f shows the alpha, beta, gamma, theta and delta rhythms of the sampled EEG signal, respectively. The alpha, beta, theta and delta rhythms were further used as suggested in [1, 14]. The wavelet packet entropy types namely “Shannon”, “Logarithmic energy”, “Threshold”, “Sure” and “Norm” and Katz and Higuchi fractal dimensions were used as features. Figure 3 shows the wavelet packet decomposition of an EEG signal. Two-level decomposition using the Daubechies wavelet function of order 4 was carried out to obtain the wavelet packet coefficients.

From Fig. 3, the first row shows the raw EEG signal, the second and the third rows show the coefficients that were obtained in the first level and the last four rows show the four coefficients that were obtained in the second level of the wavelet packet decomposition. Thus, six wavelet packet coefficients were obtained. As seven features were extracted for each wavelet packet coefficients, a total of 42 features were obtained from the wavelet packet decomposition. The “Threshold”, “Sure” and “Norm” entropy parameters were chosen as 0.2, 3.0 and 1.1, respectively. These parameters were adjusted heuristically during the experimental studies. The number of neighbors in the k-NN classifier was chosen as 3. The other numbers were also selected but the best accuracy scores were obtained when the number of nearest neighbors was 3.

The initial experiments were conducted on HV vs LV and the obtained results are given in Tables 2, 3, 4, 5 and 6, respectively. As we used 32 EEG channels, initial experiments were conducted to determine the efficient EEG channels for each of the EEG rhythms. To this end, the achievements of the EEG channels were examined and the channels that were in the top five achievements were fed into the majority voting procedure. For alpha rhythm, the obtained results were tabulated in Table 2. From Table 2, it was observed that Fp1, Fc1, T7, O1 and T8 channels produced the top five highest achievements for alpha rhythm, respectively. Table 2 also gives the related sensitivity and specificity scores. The best accuracy score of 66.25% was obtained by the O1 channel. The second-best accuracy score 62.50% was produced by the T7 channel. T8 and Fc1 channels produced the 61.25% accuracy scores and Fp1 channels produced the 60.00% accuracy score, respectively.

**Table 2:** Top 5 EEG channel achievements on alpha rhythm for HV vs LV classification

Top 5 EEG channel achievements	Sensitivity (%)	Specificity (%)	Accuracy (%)
Fp1	73.91	41.18	60.00
Fc1	58.70	64.71	61.25
T7	73.91	47.06	62.50
O1	65.22	67.65	66.25
T8	63.04	58.82	61.25
Majority voting	86.95	79.41	83.75

**Table 3:** Top 5 EEG channel achievements on beta rhythm for HV vs LV classification

Top 5 EEG channel achievements	Sensitivity (%)	Specificity (%)	Accuracy (%)
Fc5	89.13	29.41	63.75
O1	69.57	61.77	66.25
Fc6	89.13	52.94	73.75
Fp1	89.13	35.29	66.25
Cz	73.91	47.06	62.50
Majority voting	97.83	61.77	82.50

**Table 4:** Top 5 EEG channel achievements on theta rhythm for HV vs LV classification

Top 5 EEG channel achievements	Sensitivity (%)	Specificity (%)	Accuracy (%)
Fp1	67.39	50.00	60.00
F3	71.74	58.82	66.25
PO4	76.09	52.94	66.25
Fc2	80.44	55.88	70.00
Fp2	82.61	32.35	61.25
Majority voting	95.65	58.82	80.00

**Table 5:** Top 5 EEG channel achievements on delta rhythm for HV vs LV classification

Top 5 EEG channel achievements	Sensitivity (%)	Specificity (%)	Accuracy (%)
F7	71.74	44.12	60.00
F3	65.23	70.59	67.50
FC1	78.26	50.00	66.25
F4	80.44	47.06	66.25
Fp2	80.44	50.00	67.50
Majority voting	97.83	61.77	82.50

**Table 6:** The majority voting of the rhythm’s achievements for final emotion recognition (HV vs LV)

Top 5 EEG channel achievements	Sensitivity (%)	Specificity (%)	Accuracy (%)
Majority voted alpha	86.95	79.41	83.75
Majority voted alpha*	86.95	79.41	83.75
Majority voted beta	97.83	61.77	82.50
Majority voted theta	95.65	58.82	80.00
Majority voted delta	97.83	61.77	82.50
Majority voting of rhythms	100.00	67.65	86.25

The last row of Table 2 shows the results of majority voting. In majority voting, the predictions of the top 5 channels were further used to obtain a final prediction using the majority predictions of the top 5 channels. As seen in Table 2, the majority voting highly improved the classification achievement where 83.75% accuracy score was produced. This score is 17.50% better than the O1 channel’s achievement. The sensitivity and specificity scores were also highly improved.

The detected top 5 successful EEG channels for beta rhythm are given in Table 3. As seen in Table 3, Fc5, O1, Fc6, Fp1 and Cz channels obtained the better accuracy scores than the other channels on HV vs LV classification. It was observed that the best accuracy score of 73.75% was produced by the Fc6 channel. Fp1 and O1 yielded 66.25% accuracy scores. And, Fc5 and Cz produced 63.75% and 62.50% accuracy scores, respectively. When the obtained predictions were used in majority voting, 82.50% accuracy score was produced, which shows a clear improvement against the achievements of the single channel. The improvement was 8.75% on the accuracy score. Moreover, the sensitivity and specificity scores of the majority voting were 97.83% and 61.77%, respectively.

Table 4 gives the achievements of the top 5 EEG channels for theta rhythm. These channels were Fp1, F3, PO4, Fc2, and Fp2, respectively. The best accuracy score of 70.00% was produced by the Fc2 channel. Moreover, F3 and PO4 channels produced the 66.25% accuracy scores. Fp2 and Fp1 channels yielded 61.25% and 60.00% accuracy scores, respectively. Similar to the previous experiments, the majority voting of the channel achievements improved the channels of individual achievements. 80.00% accuracy score, 95.65% sensitivity, and 58.82% specificity scores were obtained with the majority voting procedure.

The performances of the top 5 EEG channels on delta rhythm are given in Table 5. The EEG channels that performed better than the other channels were F7, F3, FC1, F4, and Fp2, respectively. F3 and Fp2 channels produced 67.50% accuracy scores, which were better than the F7, FC1 and F4 channels. FC1 and F4 channels produced 66.25% the second-best accuracy scores among the top 5 channels achievements. 60.00% accuracy score was produced by the F7 channel. The majority voting procedure obtained the best accuracy score where the accuracy was 82.50%. Majority voting improved the accuracy of 15% on delta rhythm.

Finally, Table 6 shows the classification performance on HV vs LV discrimination. The majority voting predictions from all rhythms were re-used in a final majority voting procedure. As four rhythms were considered in experiments, for the final majority voting procedure, the alpha rhythm was used two times as the alpha rhythm produced the best results among all rhythms. From Table 6, it is observed that the final majority voting increased the HV vs LV discrimination. The calculated accuracy, sensitivity and specificity scores were 86.25%, 100%, and 67.65%, respectively.

Experiments were also carried out to recognize the emotions LA and HA. Tables 7, 8, 9, 10 and 11 show the obtained results, respectively. The achievements of the channels on alpha rhythm for classification of LA vs HA are given in Table 7. Fp1, Fc1, Fc6, O1, and Cp2 channels produced better achievements than the other channels. While the Fc6 channel produced 61.25% accuracy score, O1 and Cp2 channels produced 60% accuracy scores. Fc1 and Fp1 channels produced 58.75% and 57.50% accuracy scores, respectively. Majority voting improved the classification of the HA vs LA on alpha rhythm. The improvement was about 10%..

**Table 7:** Top 5 EEG channel achievements on alpha rhythm for HA vs LA classification

Top 5 EEG channel achievements	Sensitivity (%)	Specificity (%)	Accuracy (%)
Fp1	66.67	43.75	57.50
Fc1	81.25	25.00	58.75
Fc6	79.17	34.38	61.25
O1	77.08	34.38	60.00
Cp2	72.92	40.63	60.00
Majority voting	97.92	31.25	71.25

**Table 8:** Top 5 EEG channel achievements on beta rhythm for HA vs LA classification

Top 5 EEG channel achievements	Sensitivity (%)	Specificity (%)	Accuracy (%)
F7	60.42	56.25	58.75
Fc1	62.50	56.25	60.00
Fc6	64.58	50.00	58.75
Cp2	58.33	56.25	57.50
O2	95.83	12.50	62.50
Majority voting	81.25	53.13	70.00

**Table 9:** Top 5 EEG channel achievements on theta rhythm for HA vs LA classification

Top 5 EEG channel achievements	Sensitivity (%)	Specificity (%)	Accuracy (%)
F3	68.75	53.13	62.500
Fc1	75.00	40.63	61.25
Fp2	68.75	65.63	67.50
F4	85.42	37.50	66.25
Fc2	72.92	65.63	70.00
Majority voting	89.58	62.50	78.75

**Table 10:** Top 5 EEG channel achievements on delta rhythm for HA vs LA classification

Top 5 EEG channel achievements	Sensitivity (%)	Specificity (%)	Accuracy (%)
P3	79.17	40.63	63.75
Oz	66.67	43.75	57.50
Fz	62.50	53.13	58.75
C4	79.17	28.13	58.75
Cp6	93.75	21.88	65.00
Majority voting	95.83	46.88	76.25

**Table 11:** The majority voting of the rhythm’s achievements for final emotion recognition (HA vs LA)

Top 5 EEG channel achievements	Sensitivity (%)	Specificity (%)	Accuracy (%)
Majority voted alpha	97.92	31.25	71.25
Majority voted beta	81.25	53.13	70.00
Majority voted theta	89.58	62.50	78.75
Majority voted delta	95.83	46.88	76.25
Majority voted theta*	89.58	62.50	78.75
Majority voting of rhythms	93.75	71.88	85.00

Table 8 shows the performances of the top 5 channels on beta rhythm for HA vs LA classification. As seen in Table 8, the best achievement was performed by the O2 channel where the calculated accuracy score was 62.50%. Fc2 produced a 60.00% accuracy score, which was the second-best performance on beta rhythm. F7 and F6 channels produced 58.75% accuracy scores and the Cp2 channel yielded the 57.50% accuracy score, respectively. The last row of Table 8 shows the majority voting achievement on beta rhythm and 70.00% accuracy score was obtained. This score was 7.50% better than the best EEG channel.

The theta rhythm’s achievements are given in Table 9. As it was observed from Table 9, F3, Fc1, Fp2, F4 and Fc2 channels produced the top 5 accuracy scores on HA vs LA discrimination. The Fc2 channel produced a 70.00% accuracy score, which was the highest among the top 5 EEG channels. While the Fp2 channel produced the second-best accuracy score, F4, F3, and Fc1 produced the third, fourth, and fifth-best accuracy scores, respectively. The performance of the majority voting was 78.75% which was better than a single channel’s achievements. The sensitivity and specificity scores of the majority voting were 89.58% and 62.50%, respectively.

Table 10 shows the top 5 channels achievements on delta rhythm. These channels were P3, Oz, Fz, C4, and Cp6, respectively. The best accuracy 65.00% was produced by the Cp6 channel and P3 produced the 63.75% accuracy score. Fz and C4 produced 58.75% accuracy scores and the worst accuracy score of 57.50% was obtained by the Oz channel. 76.25% accuracy score was produced by the majority voting of the top 5 channels. This score showed a clear improvement against single EEG channels.

The final classification result for HA vs LA discrimination is given in Table 11. For majority voting, the predictions of the theta rhythm used twice as shown by theta and theta*. The final achievement for HA vs LA classification was 85.00%. Besides, the obtained sensitivity and specificity scores were 93.75% and 71.88%, respectively. The improvement was about 6.25%.

The performance comparison of the proposed work with some of the existing methods was given in Table 12. These methods were reviewed in the introduction section and all of them used their experiments on the DEAP dataset. When Table 12 was examined, it was seen that the proposed method outperformed other methods on HV vs LV discrimination. Alzarzi et al. produced a second-best accuracy score where the accuracy was 85.8% [3]. Abeer et al., Tripathi et al. and Li et al. reported 82.0%, 81.4% and 80.7% accuracy scores, respectively [7, 11, 14]. Rozgić, Zhang and Atkinson et al. reported accuracy scores among 70.0% and 80.0% and Zhuang, Huang and Chandra et al. reported achievements between 60.0% and 70.0% [4–6, 9, 10, 13]. The worst score 57.6% was reported by Koelstra et al. [2].

**Table 12:** Performance comparison of the proposed method with some of the state-of-the-art results

Method	Accuracy (%)	
	HV vs LV	HA vs LA
Koelstra et al. [2]	57.6	62.0
Alazrai et al. [3]	85.8	86.6
Huang et al. [4]	66.1	82.5
Chandra et al. [5]	65.1	65.3
Rozgic et al. [6]	76.9	69.1
Abeer et al. (2017)	82.0	82.0
Zhang et al. [9]	75.2	81.7
Atkinson et al. [10]	73.1	73.0
Tripathi et al. [11]	81.4	73.3
Zhuang et al. [13]	69.1	71.9
Li et al. [14]	80.7	83.7
Yin et al. [12]	83.0	84.2
Proposed study	86.3	85.0

The performance comparisons on HA vs LA discrimination are also given in Table 12. From Table 12, it was seen that Alazrai et al. reported the best classification accuracy [3]. The second-best accuracy score was also produced by the proposed method. Huang, Zhang and Li et al. reported accuracy scores among 80.0% and 85.0%, respectively [4, 7, 9, 14]. Atkinson, Tripathi and Zhuang et al. reported accuracy scores in the range of 70.0% and 75.0%, respectively [10, 11, 13]. The worst accuracy score was also reported by Koelstra et al. [2]. Yin et al. obtained 83% and 84.2% accuracy scores, respectively.

## Conclusions

In this paper, an efficient approach was proposed for EEG-based emotion recognition. The proposed method employed a two-stepped majority voting procedure to increase the performance of the emotion classification. The DEAP dataset was used in experiments and two binary classifications such as HV vs LV and HA vs LA were considered. For HV vs LV discrimination, it was seen that the alpha rhythm of the EEG signals was more convenient. Also, Fp1, Fc1, T7, O1 and T8 channels on alpha rhythm produced better accuracy scores than the other EEG channels. Beta and delta rhythms produced the 82.50% accuracy scores. For beta rhythm, Fc5, O1, Fc6, Fp1, and Cz channels performed better and for delta rhythm, F7, F3, FC1, F4, and Fp2 channels were outperformed. Theta rhythm produced the worst majority voting prediction. The second stepped majority voting produced quite improved results that the single EEG channels. The HA vs LA classification rates were lower than the HV vs LV classification rates. Theta was the best rhythm for HA vs LA discrimination and F3, Fc1, Fp2, F4 and Fc2 channels performed better than the other channels. The second stepped majority voting on HA vs LA classification improved the classification accuracy. Delta rhythm also produced the second-best majority voting prediction on HA vs LA discrimination. Alpha and beta rhythms produced similar accuracy scores. The obtained results showed that the frontal EEG channels performed well than the other EEG regions. Also, majority voting was increased the obtained accuracy scores.

In future works, the deep learning achievements will be investigated on the EEG rhythms [27–29]. Besides, the achievements of all EEG channels will be investigated in the deep learning fashion.
